# Designing universal T cell therapies: strategies to evade natural killer cells

**DOI:** 10.3389/fimmu.2026.1826738

**Published:** 2026-05-20

**Authors:** Omar Bushara, Gerald P. Linette, Beatriz M. Carreno

**Affiliations:** Center for Cellular Immunotherapies, Perelman School of Medicine, University of Pennsylvania, Philadelphia, PA, United States

**Keywords:** CAR-T, HLA-E, NK cells, TCR-T, universal T cells

## Abstract

Cell therapies such as chimeric antigen receptor (CAR) T cells and T cell receptor (TCR) T cells marked transformative advances in the treatment of hematologic and solid malignancies, respectively. Thus, adoptive T cell therapy (ACT), in which autologous T cells sourced from the patient constitute the starting immune population, represents a contemporary modality for the treatment of cancers. The need of an autologous cell product poses scientific and logistical challenges that need to be overcome to develop efficacious, scalable and cost-effective ACT. Peripheral blood lymphocytes procured from healthy donors can serve as a starting population for manufacturing a universal allogeneic T cell product offering solutions to both challenges. Recent advances in gene-engineering and -editing technologies have facilitated progress in the development and large-scale manufacturing of allogeneic T cell products. A strategy in development of allogenic ACT is ablation of the TCRαβ/CD3 complex to avoid graft versus host disease mediated by unrelated donor T cells. Mitigating host allogeneic T cells recognition is a complex endeavor that may begin with HLA-I/-II ablation, avoiding recognition and rejection of “non-self” HLA molecules. However, HLA-deficient T cells are susceptible to host NK cell recognition via the “missing-self” response. Here, we discuss immune evasive strategies taken to reduce NK cell mediated rejection of HLA-deficient T cells with particular emphasis on exploitation of HLA-E, a non-classical HLA-I, with regulatory function on NK cell activity. Current progress suggests that off-the-shelf universal T cell products may evolve to become a standard of care treatment options for certain disease indications.

## Introduction

The advent of immunotherapy represents a significant advancement in effective treatment for cancer (1, 2). Immunotherapy has evolved in parallel with an increased understanding of the human immune system and the subsequent development of novel monoclonal antibodies, cytokines, biological modifiers, and cellular therapies (3). A particularly promising modality of immunotherapy for cancer has more recently emerged – gene modified autologous T cells such as chimeric antigen receptor (CAR)-T cell, and T cell receptor-engineered (TCR)-T cell therapy. CAR-T cells received initial regulatory approval for acute lymphoblastic leukemia and diffuse large B cell lymphoma in 2017 (4, 5). TCR-T cells are a more recent development and received FDA approval for the treatment of synovial sarcoma in 2024 (6).Although promising, there are limitations with current autologous T cell therapies. The process of harvesting and manipulating patient T cells is resource-intensive and thus restricted to a few large academic centers and industry (7, 8). Additionally, the current manufacturing time may be as long as 4 weeks, which limits access for patients with rapidly progressive disease who require more urgent intervention. Patient-derived T cells may be depleted of naïve/early memory populations or be dysfunctional due to receipt of previous therapies such as cytotoxic chemotherapy, which ultimately limits the effectiveness of many autologous cell products. Age is a final consideration as immune senescence is a prominent aspect of the functional decline observed in various innate and adaptive compartments of the elderly human immune system (9). Thus, utilization of allogeneic cells harvested from healthy donors may offer the ideal source of effector T cells. This approach would entail harvesting normal healthy donor T cells and manufacturing an allogeneic cell product to provide an “off-the-shelf,” or universal, cellular immunotherapy which could be administered to eligible patients without regard to histocompatibility barriers (10–13). Although this approach would broaden the patient population eligible for treatment and has obvious appeal, there are inherent limitations related to histocompatibility differences using cells from unrelated donors.

Several solutions have been employed to enable the genetically modified unrelated donor T cells to evade the host’s immune system. First, host lymphodepletion is often employed in order to reduce the potential for rejection. This is traditionally accomplished by chemotherapy, and specifically with the combination of fludarabine and cyclophosphamide, however recent methods utilize anti-CD52 therapy (alemtuzumab) which depletes mature T cells implicated in allo-rejection (14–18). An alternative solution is the ablation of HLA-I and HLA-II surface molecules on donor T cells to avoid the recognition by host T cells as “non-self.” However, ablation of HLA class I makes donor T cells vulnerable to the natural-killer cell-mediated “missing-self” response. This represents a major hurdle in allogeneic cell therapy, and thus strategies for evasion of NK cell-mediated rejection are of interest. One promising strategy is the overexpression of HLA-E, a non-classical HLA molecule that can provide inhibitory signals to NK cells in the appropriate context. Recently, our group demonstrated that CAR-T cells enhanced with expression of a HLA-E-β2m-peptide single-chain trimer avoided NK cell-mediated rejection in a pre-clinical tumor model (19).

In this review, we describe approaches for engineering allogeneic/universal adoptive T cells to evade elimination by host NK cells. Further, we highlight HLA-E as a promising pre-clinical strategy employed by our group and others. Examples of strategies described herein is found in **Table 1**.

**Table 1:** Examples of strategies employed to facilitate allogenic T cell therapies.

Strategy	Stage of evidence	References	T cell product target	Limitations
Strategies to Prevent T Cell-Mediated Rejection				
TCR/CD3 ablation	Clinical (Phase I/II)	Locke et al, 2025 (PMID 39946666) NCT03939026	CD19 (cema-cel), CD7, BCMA	Residual TCR+ cells remain
HLA-I ablation (β2m KO)	Clinical (multiple trials)	Kagoya et al, 2020 (PMID 32321775) NCT05722418	CD19, CD22, BCMA, CLL-1	Triggers NK missing-self response
CD52 KO + alemtuzumab lymphodepletion	Clinical (Phase I)	Locke et al, 2025 (PMID 39946666) NCT03939026	CD19 (cema-cel), CD7 (BE-CAR7)	Non-specific immunodepletion and resultant morbidity, NK recover faster than T cells after depletion
Host lymphodepletion (fludarabine/cyclophosphamide)	Clinical (standard of care)	Locke et al, 2025 (PMID 39946666)	All allogeneic products	Non-specific immunodepletion and resultant morbidity
ADR — CD7	Clinical (Phase I)	Chiesa et al, 2023 (PMID 37314354) Pan et al, 2021 (PMID 34324392)	CD7 T-ALL (BE-CAR7)	T cell aplasia, currently restricted to CD7+ malignancies, requires CD7 KO
ADR — CD70	Clinical (Phase I)	Wang et al, 2025 (PMID39793572) NCT07085104 (RESOLUTION trial)	CD19/CD70 dual (ALLO-329)	CD70 widely expressed on activated immune cells
HLA-II ablation (CIITA KO)	Preclinical	Kagoya et al, 2020 (PMID 32321775)	CD19	Incomplete protection in isolation
FAS KO	Preclinical	Menegatti et al, 2024 (PMID 39558141)	CD19	Partial protection in isolation
ADR — 4-1BB/OX40	Preclinical	Mo et al, 2021 (PMID 32661440)	CD19, multi-target	Only addresses activated T cells
Strategies to Prevent NK Cell-Mediated Rejection				
MICA/B downregulation (NKG2D ligand ablation)	Clinical (Phase I)	Pollyea et al, 2025 (PMID 40954213) NCT04167696 (CYCLE-1 trial)	NKG2DL-targeted (CYAD-02)	Removes activating NKG2D ligand only, incomplete NK protection in isolation
SPPL3 KO (glycan shielding)	Clinical (Phase I)	Wu et al, 2025 (PMID 40845838) NCT06014073	CD19 (TCR-KO and TCR-sufficient variants)	Limited persistence
CD47 overexpression	Clinical (Phase I)	Hu et al, 2025 (PMID 40812299) NCT05878184 (SC291 ARDENT trial)	CD19 (SC291)	Primarily inhibits myeloid rejection, limited direct NK inhibition
HLA-E overexpression	Clinical (Phase I)	Li et al, 2022 (PMID 36532006) NCT05722418 (CaMMouflage)	BCMA (CB-011)	Inhibits only NKG2A+ NK cells, NKG2C+/A- NK cells may become activated
HLA-G overexpression	Preclinical	Guo et al, 2021 (PMID 34323289)	CD19, iPSC-derived products	Partial protection as LILRB1/ILT2 expressed only on NK cell subsets
CD300a TASR (trans-antigen surface receptor)	Preclinical	Zhang et al, 2025 (PMID 39368806)	Multi-target	Variable CD300a expression across NK and T cells
CD155/CD112 ablation (DNAM-1 ligand ablation)	Preclinical	Hammer et al, 2024 (PMID 38981470)	Multi-target	Removes DNAM-1 activating signals only, incomplete NK protection in isolation
B7-H6 ablation (NKp30 ligand ablation)	Preclinical	Kilian et al, 2024 (PMID 38701193)	Multi-target	Removes NKp30 activating ligand only, incomplete NK protection in isolation
CD54/CD58 ablation (NK-T cell adhesion disruption)	Preclinical	Hammer et al, 2024 (PMID 38981470)	Multi-target	Impairs NK-T cell adhesion only, incomplete NK protection in isolation
Nef expression (viral immune evasion)	Preclinical	Perica et al, 2025 (PMID 39884316)	CD19	Partial HLA-I downregulation only, residual HLA-I may still lead to rejection and HLA-I ablated cells may trigger NK missing self response

## Allogeneic barriers to universal T cell therapies

Alloreactivity represents a primary barrier to universal T cell therapies (20–22). Alloreactivity mediated by donor T cell recognition of non-self HLA molecules on host (recipient) tissues may cause Graft versus Host Disease (GvHD) resulting in mild-severe morbidity especially in immunosuppressed individuals (21, 22). As such, genetic ablation of the αβ T cell receptor (TCR) or CD3 subunit components of donor T cells removes the endogenous TCR cell surface expression, reducing alloreactivity and the risk of GvHD. For current CAR-T cell product designed with scFv, gene editing approaches have been developed using CRISPR-cas9 or base editors to ablate the endogenous TCR α or β chains, or alternatively, CD3 subunit components, with high efficiency thereby mitigating the risk of GvHD in patients (23–26).

Despite the ablation of endogenous TCR, donor CAR-T and TCR-T cells that express HLA-I and HLA-II molecules are inevitably eliminated in an immunocompetent host due to the Host versus Graft (HvG) response. A strategy to mitigate host alloresponses responses to donor CAR-T cells is knockout of *FAS*, encoding for T cell expression of Fas (CD95) cell surface death receptor. CD95 is classically associated with an alternative cytotoxicity mechanism upon engagement with FasL (CD95L), and was identified in a pre-clinical study as modulating alloreactivity in adoptive T cells (27, 28). In this study, ablation of CD95 in CAR-T cells yielded improved persistence while avoiding both host T cell-mediated rejection and NK cell sensitization (27, 28). Another more recent approach to prevent HvG response is engineering T cells that express not only an antigen-specific receptor (CAR or TCR), but also an alloimmune defense receptor (ADR) which recognizes a receptor/ligand on activated lymphocytes. This ADR has a CD3ζ chain intracellular domain that results in clearance of the activated host lymphocytes that mediate rejection. Importantly, ADRs do not recognize naïve (or resting) lymphocytes and thus allow evasion of alloreactivity without widespread elimination of most host peripheral blood lymphocytes. ADR systems using CAR-T cells that recognizes 4-1BB, OX40, CD7, or CD70, known surface markers that are expressed on activated T cells. For CD7 and CD70, ablation of these markers on the engineered allogenic T cells should be considered to avoid fratricide. These ADR-expressing CAR-T cells clear both tumor cells and activated lymphocytes, while sparing resting lymphocytes (29–34).

Genetic ablation of HLA-I/II molecules, using TALEN, CRISPR-cas9 or base-editing, has been proposed as another strategy to avoid host rejection and ensure persistence of allogeneic CAR-T and TCR-T cells (35–37). HLA-I molecules are present on all nucleated cells and are composed of the non-polymorphic β2-microglobulin (β2m) and a highly polymorphic heavy alpha chain which together with peptides form tripartite complexes encoding HLA-A, B, C (HLA-Ia, classical) molecules. Similar tripartite complexes are formed by HLA-E, F, G (HLA-Ib, non-classical) molecules, although these heavy alpha chains exhibit limited polymorphism. The most common approach to downregulate HLA-I from the donor T cell surface is to genetically ablate expression of β2m, effectively eliminating HLA-I class Ia and class Ib expression. HLA-II molecules are upregulated in activated human T cells and thus also present an allogenic barrier to donor T cell engraftment (35–38). To globally downregulate all HLA-II (DR, DP, DQ) molecules, the most common approach is to target the master regulator of HLA-II expression, MHC class II Transactivator Complex (CIITA) (23, 35–39). However, ablation of HLA-I on the T cell surface triggers the NK cell-mediated “missing-self” response. NK cell-mediated rejection is thus a significant barrier to effective HLA-I/II ablated/allogenic T cell therapy.

## The NK “missing-self” challenge

Unlike CD8+ T cells, which are activated through engagement of their TCR with HLA-I/peptide complexes, NK cell activation is regulated by the integration of signals delivered by inhibitory receptors, including NK killer Ig-like (KIR) receptor and the NKG2A/CD94 complex, and activating receptors, including activating KIRs, NKG2C/CD94 complex, NKG2D, NKp30 and others (40–42). NK Inhibitory KIRs and the NKG2A/CD94 receptor interaction with HLA-Ia molecules induces a dominant “tolerogenic/inhibitory” signal. Downregulation of HLA-Ia expression by pathogens and tumors to evade CD8+ T cell responses, leads to a NK “missing-self” response due to loss of inhibitory receptor engagement. Thus, this “missing-self” response serves as a sensor for NK cellular responses potentiating their cytotoxicity and secretion of inflammatory cytokines such as IFNγ and TNFα (43). However, this critical and necessary “missing-self” response presents a major hurdle limiting the potential of adoptive cell therapy employing HLA-I/II ablated allogeneic CAR-T and TCR-T cells. This hurdle is further complicated by the heterogeneity of inhibitory and activating receptor expression on NK cell populations. This NK diversity is observed both within and between individuals, thus making it a challenge to favor a tolerogenic/inhibitory signal (44, 45).

## HLA-E as a modulator of NK cell activity

**Figure 1:**
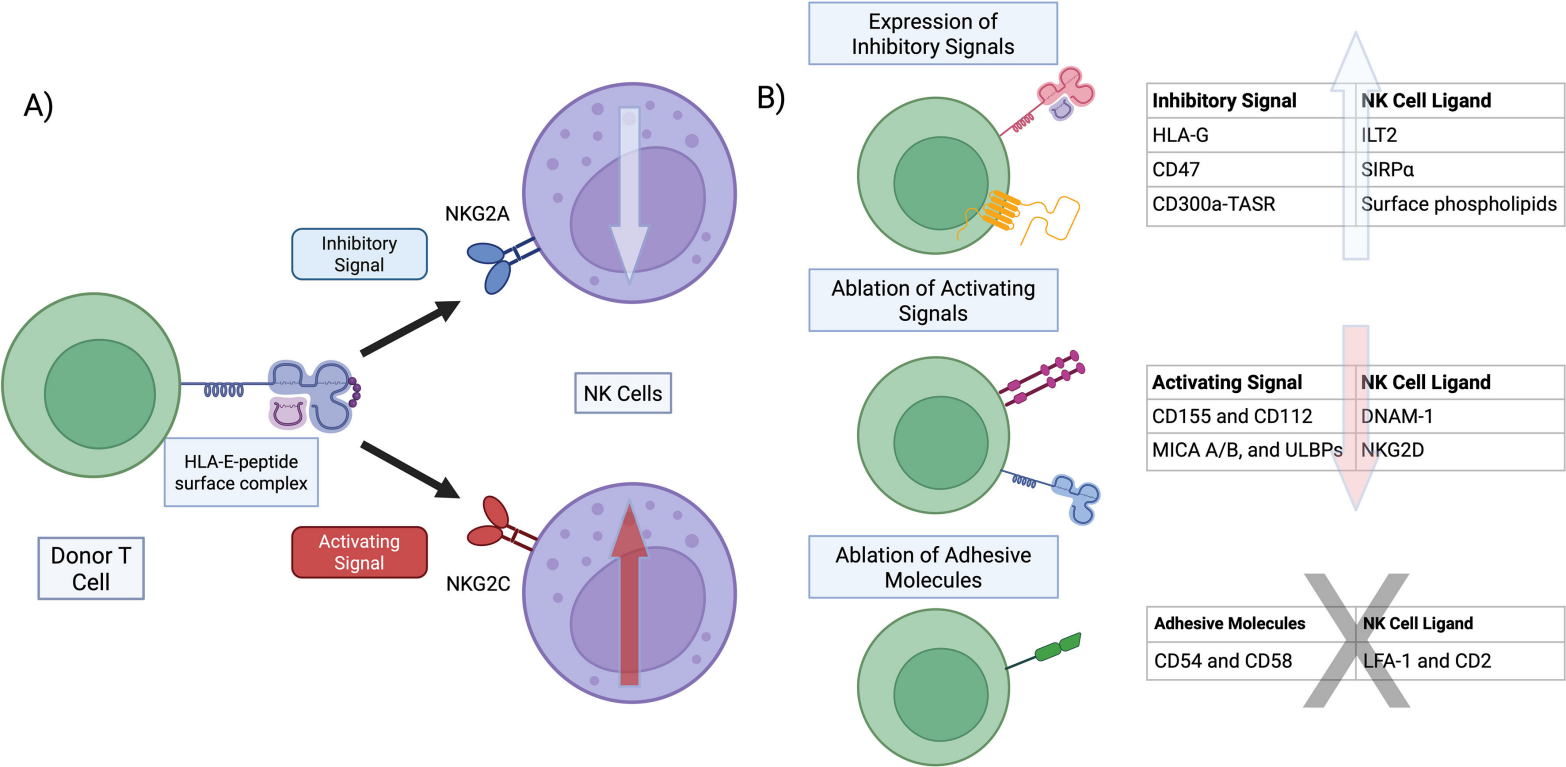
Strategies to promote NK cell evasion **(A)** HLA-E interaction with the CD94/NKG2x complexes can deliver inhibitory or activating signals to NK cells. These signals are delivered through an immunoreceptor tyrosine-based inhibitory motif (ITIM) in NKG2A/CD94 and an immunoreceptor tyrosine-based activating (DAP12/ITAM-coupled) in NKG2C/CD94. Peptides presented by HLA-E may favor interaction with NKG2A or NGK2C, tipping the balance in favor of an inhibitory or activating signal, respectively. **(B)** Other strategies for evasion of NK cell-mediated rejection include overexpression of other inhibitory signals, ablation of activating signal receptors, and ablation of surface molecules involved in T cell-NK cell adhesion. The various receptors or ligands that fall in these categories are listed. Created with BioRender.com.

HLA-E, a non-classical HLA-class Ib molecule, is a key regulator of NK cell activity, and thus its expression represents a potential tool for evading NK cell-mediated clearance. In the population, HLA-E presents 2 predominant alleles, E*01:01 and E*01:03, that differ by a single amino-acid at position 107, Arginine at E*01:01 and Glycine at E*01:03. Although these amino acid differences are outside the peptide binding groove, this dimorphism affects HLA expression with E*01:03 exhibiting higher cell surface expression than E*01:01 (46). Further, HLA-E is unique in the repertoire of peptides it binds and presents at the cell surface. Most HLA-E presented peptides consist of nonamers and exhibit a conserved AA sequence motif with Valine at position 1 and Leucine at position 9, so called “VL9” peptides (47). These VL9 peptides are primarily derived from signal peptide sequences of classical HLA-Ia alleles generated through peptidases and proteosome processing (48, 49). Only a select number of HLA-I alleles generate VL9 peptide variants that can be processed and bind to HLA-E with sufficient affinity to promote tripartite HLA-E-β2m-peptide complex formation and cell surface expression (47). These tripartite HLA-E complexes have a rapid cell surface turnover and modulate, through the presentation of “self” classical HLA-I derived VL9 peptides, NK cell responses by engaging with NKG2A/CD94 and NKG2C/CD94 complexes on NK cells (47, 50)(**Figure 1A**).

NKG2A/CD94 and NKG2C/CD94 are C-type lectins expressed on NK cells that, upon binding to tripartite HLA-E-β2m-peptide complexes deliver inhibitory or activating signals, respectively. Such signals are delivered through an immunoreceptor tyrosine-based inhibitory motif (ITIM) in NKG2A/CD94 and an immunoreceptor tyrosine-based activating (DAP12/ITAM-coupled) in NKG2C/CD94. Expression of these receptors is heterogeneous among NK cell populations, with only a small percentage of cells expressing both receptors. Additionally, individuals infected with CMV and other pathogens, present variable percentages of NKG2C+ NK cells, so called adaptive or memory NK cells. NKG2A/CD94 receptors display a higher affinity for tripartite HLA-E-β2m-peptide complexes than NKG2C/CD94, resulting in dominant inhibitory signals. Additionally, HLA-E presented peptides may deliver distinct signals may thru NKG2A and C receptors. While those thru NKG2A/CD94 are largely inhibitory, AA composition of presented peptides are critical in determining stimulatory signals delivered thru NKG2C/CD94 (51). Importantly, this feature is exploited by viruses such as CMV and HIV, with HLA-Ia expression downregulated to evade CD8+ T cell killing of virus-infected cells, while HLA-E expression maintained to promote inhibition of NK killing (52, 53). These observations suggest defined tripartite HLA-E-β2m-peptide complexes may represent an attractive “armoring” strategy to modulate the NK missing-self response toward HLA-I/II ablated allogeneic cell products.

Given its putative role as an NK cell modulator, expression of synthetic HLA-E- β2m dimers or HLA-E-β2m-peptide single-chain trimers has been employed as a strategy to evade NK cell reactivity in HLA-I (β2m)-ablated T cells and pluripotent stem cells. Our group has employed this strategy in a pre-clinical model of anti-CD19 CAR-T cells that express an HLA-E single-chain trimer. We demonstrated significantly reduced NK cell-mediated lytic activity and improved persistence of HLA-I/II ablated allogeneic T cells. This strategy has been shown to be effective by others, as well, and demonstrates reduced *in vitro* and *in vivo* CAR-T cell clearance in a variety of tumor models (19, 38, 54–56). There is an ongoing trial utilizing HLA-E armoring in the setting of anti-BCMA CAR-T cells in multiple myeloma (NCT05722418), building on a successful pre-clinical model by the same group (57).

Of consideration for future trials is patient-to-patient variation in NK cell phenotype. As mentioned above, interaction of HLA-E with NKG2A is inhibitory while with NKG2C is activating. Importantly, NKG2A+/C-, NKG2A-/C+, and NKG2A+/C+ cells are all possible NK populations found within patients. Thus, the broader NK cell phenotype within enrolled patients should be considered – patients who are NKG2A+/C- would derive the most benefit from HLA-E armoring, while patients with NKG2A-/C+ NK cell repertoires receive diminished protection. Patients who are NKG2A+/C+ would derive benefit, as the inhibitory signal is dominant. Although promising in the right patient population, this limitation in NKG2C dominant patients merits exploration of other strategies able to reduce NK cell-mediated rejection.

## Other strategies facilitating universal adoptive T cells

The overexpression of HLA-E is one of several strategies posited to protect universal engineered T cells against NK cell attack. Herein, we present other approaches that allow for evasion of NK cell-mediated recognition (**Figure 1B**).

## Delivering inhibitory signals to NK cells

### HLA-G

HLA-G is a non-classical MHC I molecule with suppressive immunoregulatory functions. HLA-G has been shown to be tolerogenic and is well-studied in the context of maternal-fetal immune reactivity in pregnancy (58–60). HLA-G interacts with the receptor ILT2, expressed on NK cells and memory immune cells, and has been shown to inhibit maternal immune response to fetal antigens (60, 61). As such, overexpression of HLA-G may be leveraged similarly to the overexpression of HLA-E. Direct comparison of HLA-E and HLA-G expression to inhibit NK cells attack of β2m-deficient Jurkat T cells was reported, and both constructs appear to be partially protective (54).

### CD47

Overexpression of CD47 has also been shown to inhibit NK cell-mediated cytotoxicity. CD47 is a ubiquitously expressed transmembrane protein and binds to signal regulatory protein α (SIRPα) which is expressed on macrophages and dendritic cells, often referred to as the “don’t eat me” signal (62, 63). Recent studies have suggested that SIRPα may also be expressed on NK cells, and that binding to CD47 similarly yields an inhibitory signal (64). As this SIRPα-CD47 axis reduces cytotoxic NK cell activity, it has been implicated in immune escape and reduced anti-tumor immunity (65). Although problematic in that context, harnessing this axis by engineering cells that overexpress CD47 may reduce myeloid cell- and NK cell-mediated rejection of universal cell products in patients. Pre-clinical testing of the allogeneic TRAC/β2m/CIITA gene-edited/CD47+ modified CART19 cells demonstrated persistence with anti-tumor activity in fully immunocompetent allogeneic humanized NSG animals in the Nalm6 leukemia model (66). Recent pilot data in patients with hypo-immune gene-edited/engineered allogeneic CART19 cell product confirms persistence with efficient depletion of peripheral blood CD19+ cells suggesting evasion of the host T and NK cell response (67).

### CD300a trans-antigen surface receptor

CD300a is an inhibitory immune checkpoint molecule found on many hematopoietic cells, including NK cells. CD300a binds to phosphatidylserine and phosphatidylethanolamine which are phospholipids on stressed and dying cells (68, 69). A recent report suggests an engineered CD300a agonist ligand (such as scFv specific for CD300a) expressed on Universal T cells – may engage with host NK and myeloid cells to downregulate their activity, thereby mitigating potential rejection (70). In this study, this CD300a TASR was shown to outperform several alternative strategies demonstrating the potential of this approach, however continued research is warranted on this approach.

## Ablating activating signals and perturbation of the NK cell-T cell immune synapse

### Ablation of NK activating ligands

DNAM and NKG2D are well studied activating receptors on NK cells. Ligands for DNAM-1 include CD155 and CD112, both of which are found on CD8+ T cells, while ligands for NKG2D include the MHC class I-related proteins (MICA/B) and the UL-16 binding proteins (ULBPs) (71, 72). While there is limited data, studies implicate both CD155 and CD112 as potential ligands that serve to promote recognition and lysis of Universal CAR T cell by NK cells (71). Similarly, a recent study demonstrated improved persistence and efficacy of autologous CAR-T cells engineered with shRNA to downregulate MICA/B (73). Finally, B7-H6 expressed by activated CAR T cells was recently described as a novel ligand which binds NKp30 (74, 75). In one study, gene ablation of B7-H6 promoted the persistence of CAR-T cells in the presence of NK cells both *in vitro* cultures as well as NALM6 bearing NSG mice reconstituted with human NK cells (75). Together, these observations support gene-editing of CAR T cells focused on the deletion of ligands that activate NK cells.

### Ablation of CD54 and CD58

Another strategy is to impair the ability of NK cells to engage with and adhere to Universal allogeneic T cells. Two well-known adhesion ligands expressed on T cells are CD54 and CD58 which interact with their respective receptors, LFA-1 and CD2 on NK cells, promoting NK cell activation (76, 77). Deletion of CD54 and/or CD58 on allogeneic T cells has been shown to reduce NK-mediated cytotoxicity which correlated with improved T cell persistence (78).

## Additional strategies

### Cell surface glycosylation via genetic knockout of SPPL3

Modifying the proteoglycan cell surface milieu of CAR T cells has been proposed as one way to avoid recognition by host NK cells. It has been recently demonstrated that the accumulation of glycans on the cell surface leads to reduced recognition and diminished cytotoxicity by NK cells (79). A key regulator of the accumulation of glycans at the cell surface is the intramembrane protease signal peptide peptidase-like 3 (SPPL3), which cleaves proteoglycans adherent to the cell membrane. As such, knockout of SPPL3 leads to accumulation of glycans which, in turn, obscures epitopes normally recognized by NK cells (80, 81). A recent trial of glycan-shielded CAR-T cells in patients with relapsed or refractory lymphoma demonstrated that SPPL3 knockout does promote T cell persistence while maintaining CAR-T cell functionality (82).

### Leveraging viral immune evasion strategies – Nef expression

Harnessing viral immune evasion mechanisms for the purpose of reducing alloreactivity is an emerging strategy. The anti-viral immune response shows significant parallels with the alloreactive response, and as such, viral immune-evasion may be applied in the context of Universal T cell therapy (83). These strategies center around modulation of MHC molecules and cellular machinery involved in antigen presentation (83). A specific method initially described in viruses is the HIV-1 protein Nef, which has been shown to downregulate MHC-I on the infected T cell surface, thus contributing to latent viral reservoirs in T cells that escape immune recognition (84). This has been recently applied to CAR-T cells where expression of Nef resulted in reduction, but not complete loss, of HLA-I, thus reducing alloreactivity but also avoiding the NK-cell “missing self” response. Notably, Nef expression also inhibits apoptosis by activating pro-survival serine kinase Pak2, thereby increasing CAR-T persistence (85).

## Future directions

Many patients treated with adoptive T cells either fail to respond or relapse. Allogeneic universal cell products may allow the selection of specific T cell populations from healthy donors for manufacturing, potentially providing for T cell therapies with improved functionality and persistence, and thus better outcomes (86). To date, allogeneic products in published clinical trials have not fully addressed the critical hurdle for universal adoptive T cell therapy – the “missing-self” response and host NK-mediated rejection. Multiple strategies may be necessary to overcome this challenge, and it is likely to require combinations of the methods discussed above. Testing of these combinations will require development of sophisticated humanized mouse models reconstituted with allogeneic human T cells and NK cells. Additionally, novel ways to allow for precise lymphodepletion of T cells that mediate allorejection, and thus avoidance of the morbidity incurred by chemotherapy, are needed. As mentioned, anti-CD52 therapy represents a promising step towards that goal. Finally, development of *in vivo* T cell engineering approaches, utilizing either lipid nanoparticles carrying mRNA or viral vectors with integrating payloads, may successfully bypass ex-vivo T cell manufacturing and the requirements for patient conditioning, and may represent alternative strategies to allogeneic universal adoptive T cell therapy (87). However, both *in vivo* strategies have challenges of tissue and cell selectivity, and further research is warranted prior to their widespread uptake. Safety profiles are additional hurdles to be addressed by *in vivo* engineering. In sum, ex-vivo gene-engineering methodologies in allogeneic cells, whether T cells, iPS and other cells, offer tractable adoptive T cell products with increasing feasibility in the immediate future and are poised to offer life-saving therapies to a broader patient population.
